# Advancing arabic dialect detection with hybrid stacked transformer models

**DOI:** 10.3389/fnhum.2025.1498297

**Published:** 2025-02-11

**Authors:** Hager Saleh, Abdulaziz AlMohimeed, Rasha Hassan, Mandour M. Ibrahim, Saeed Hamood Alsamhi, Moatamad Refaat Hassan, Sherif Mostafa

**Affiliations:** ^1^Faculty of Computers and Artificial Intelligence, Hurghada University, Hurghada, Egypt; ^2^Insight SFI Research Centre for Data Analytics, School of Engineering, University of Galway, Galway, Ireland; ^3^Atlantic Technological University, Letterkenny, Ireland; ^4^Computer Science Department College of Computer and Information Sciences, Imam Mohammad Ibn Saud Islamic University (IMSIU), Riyadh, Saudi Arabia; ^5^Department of Computer Science, Faculty of Science, Aswan University, Aswan, Egypt; ^6^Information Technology Department, College of Computer and Information Sciences, Imam Mohammad Ibn Saud Islamic University (IMSIU), Riyadh, Saudi Arabia; ^7^Department of Computer Science and Engineering, College of Informatics, Korea University, Seoul, Republic of Korea

**Keywords:** Arabic dialects, Bert-Base-Arabertv02, Dialectal-Arabic-XLM-R-Base, transformer, Knowledge representation, NLP, deep learning, stacking model

## Abstract

The rapid expansion of dialectally unique Arabic material on social media and the internet highlights how important it is to categorize dialects accurately to maximize a variety of Natural Language Processing (NLP) applications. The improvement in classification performance highlights the wider variety of linguistic variables that the model can capture, providing a reliable solution for precise Arabic dialect recognition and improving the efficacy of NLP applications. Recent advances in deep learning (DL) models have shown promise in overcoming potential challenges in identifying Arabic dialects. In this paper, we propose a novel stacking model based on two transformer models, i.e., Bert-Base-Arabertv02 and Dialectal-Arabic-XLM-R-Base, to enhance the classification of dialectal Arabic. The proposed model consists of two levels, including base models and meta-learners. In the proposed model, Level 1 generates class probabilities from two transformer models for training and testing sets, which are then used in Level 2 to train and evaluate a meta-learner. The stacking model compares various models, including long-short-term memory (LSTM), gated recurrent units (GRU), convolutional neural network (CNN), and two transformer models using different word embedding. The results show that the stacking model combination of two models archives outperformance over single-model approaches due to capturing a broader range of linguistic features, which leads to better generalization across different forms of Arabic. The proposed model is evaluated based on the performance of IADD and Shami. For Shami, the Stacking-Transformer achieves the highest performance in all rates compared to other models with 89.73 accuracy, 89.596 precision, 89.73 recall, and 89.574 F1-score. For IADD, the Stacking-Transformer achieves the highest performance in all rates compared to other models with 93.062 accuracy, 93.368 precision, 93.062 recall, and 93.184 F1 score. The improvement in classification performance highlights the wider variety of linguistic variables that the model can capture, providing a reliable solution for precise Arabic dialect recognition and improving the efficacy of NLP applications.

## 1 Introduction

Dialects within a language are crucial as they represent the various cultural and regional variances within that language (Gregory and Carroll, 2018). As languages change and spread over different geographic areas, dialects naturally arise. Dialects may have their idiomatic phrases, distinct vocabulary, syntax, and pronunciation. Learning dialects has multiple benefits, including better communication, a greater understanding of culture, potential for employment, and increased interaction with media and literature (Zhang and Hansen, 2018). It makes it more straightforward to comprehend the variety within a language and makes it easier to build genuine connections with individuals from various geographical areas (Samih, 2017).

Given the large geographic area in which Arabic is spoken, dialects are essential for the Arabic language. Arabic dialects vary considerably from Modern Standard Arabic (MSA), the standard form for the language (Zaidan and Callison-Burch, 2014). Understanding the regional slang, customs, and traditions specific to each Arabic dialect is possible through understanding dialects. This improves comprehension of culture and makes handling social situations easier. Being fluent in a particular dialect pertinent to your line of work can help you get better employment and more significant support to Arabic-speaking communities (Alosaimi et al., 2024).

Gather a wide range of Arabic language samples across several dialects. The relevant dialect information needs to be labeled on the dataset. The data should be preprocessed by dividing it into training, validation, and test sets, tokenizing the text, and turning it into numerical representations (Haque et al., 2018). Learn a transformer model to identify dialects in Arabic. After the input text has been tokenized, the model should be able to predict the dialect label. Dialect identification requires contextual information captured by the transformer's self-attention mechanism (Lin et al., 2020). The labeled dataset is used to train the model employing optimization techniques (Chapelle et al., 2008).

Deep Learning (DL) and Machine Learning models (ML) have demonstrated promise in processing complicated linguistic data and dialects of Arabic. For example, Elaraby and Abdul-Mageed (2018) applied different ML models: SVM, RF, NB, and LR. Alzu'bi and Duwairi (2021) applied Recurrent Neural Networks (RNN) to support multiple classes of dialects. Alansari (2023) analyzed characteristics of dialects using CNN and RNN. Other authors proposed a hybrid model such as CNN-RNN (Abdelazim et al., 2022). These studies used classical DL models, which cannot capture the long-term dependencies over long sequences.

Therefore, the transformer model has attention features that allow the model to focus on the most relevant parts of the input sequence, capturing long-range dependencies and complex relationships between words (Zhang et al., 2019; Hafiz et al., 2021). For example, Alghamdi et al. (2022) applied two transformer models, MARBERT and ARBERT, using two publicly available Arabic Online Commentary (ADC) (Elaraby and Abdul-Mageed, 2018). In our work, we use recent IADD datasets that were combined from datasets such as (ADC), Dialectal ARabic Tweets dataset (DART) (Alsarsour et al., 2018), the authors in Alghamdi et al. (2022) and Elaraby and Abdul-Mageed (2018) used AOC dataset is published at 2018, and is a subset of IADD, and do not apply stacking model. As a result, the novelty of this paper lies in the combination of transformer models and a meta-learner in a stacking framework designed for Arabic dialect classification. The proposed hybrid model greatly improves the state-of-the-art Arabic dialect detection, outperforms conventional methods, and captures a greater range of linguistic features.

### 1.1 Motivations and contributions

The motivation behind the paper is the increasing amount of dialectal Arabic information produced by social networks and the need to improve Natural language processing (NLP) functions such as knowledge representation and machine translation. NLP faces challenges due to the fast expansion of dialectal Arabic material on social networks. Substantial language disparities between Arabic dialects and Modern Standard Arabic (MSA) present serious challenges for current NLP models, while this rise provides a wealth of resources for linguistic and computational study. Critical NLP applications like knowledge representation, sentiment analysis, and machine translation are hampered by the models' frequent difficulties with accurate classification and generalization across languages. Classical DL models: CNN, GRU, and LSTM have demonstrated promise in processing complicated linguistic data. Still, these techniques cannot adequately capture the subtle and nuanced differences across Arabic dialects. Furthermore, a significant research vacuum restricts NLP models' wider usability and resilience in Arabic contexts due to the absence of customized solutions to handle these dialectal variances.

To address this gap, we propose a novel stacking model that combines a meta-learner with two transformer architectures: Bert-Base-Arabertv02 and Dialectal-Arabic-XLM-R-Base. By collecting a wider variety of linguistic variables, the proposed models improve dialect categorization, performance, and generalization across different Arabic dialects. Improved classification accuracy, useful applications in machine translation, sentiment analysis, conversational AI, and a strong framework that can be modified to operate with additional low-resource or linguistically challenging languages are some of the added values. The contributions improve the usability and effectiveness of NLP systems for Arabic-speaking regions. The proposed model delivers better performance across different Arabic dialects, increased generalization, and superior dialect classification by integrating various linguistic characteristics. The main contributions of this paper are summarized as follows:

- We introduce a novel stacking model that incorporates two transformer architectures, Bert-Base-Arabertv02 and Arabic-XLM-R-Base, as base models with combined Random Forest (RF) as a meta-learner to enhance classification. The proposed model performs more efficiently than the state-of-the-art models, including LSTM, GRU, CNN, and two transformer models.
- We evaluate the proposed model performance across two datasets to demonstrate the performance in classifying four and five Arabic dialects. Stacking-Transformer has the highest performance in all rates compared to other models.
- The combination of Transformer in stack modeling with a meta-learner helps to capture more linguistic features, enhance generalization, and accurate dialect detection of Arabic.

### 1.2 Paper structure

The remainder of the paper is organized into sections. Section 2 presents related works on Arabic dialects. Section 3 outlines the primary steps for classifying Arabic dialects and introduces the proposed model. Section 4 presents the results and discussion, followed by the conclusion in Section 5.

## 2 Related work

This section presents different researcher have been applied DL, ML, and transformer models to classify Arabic dialects.

Lulu and Elnagar (2018) recognized dialects in Arabic using Four DL models CNN, LSTM, Bidirectional LSTM (Bi-LSTM), and Convolutional LSTM (CLSTM). The authors made use of the Arabic Online Commentary (AOC) dataset, which classifies Arabic into three main dialects: Gulf (including Iraqi), Levantine (LEV), and Egyptian (EGP). LSTM produced the most accurate results. Alsaleh and Larabi-Marie-Sainte (2021) utilized Genetic Algorithms (GA) to optimize the parameters of CNN for Arabic Text Classification. GA was employed to tackle the challenge of randomly initialized weights in CNN. The study utilized two extensive datasets that support text classification. Various pre-processing steps were applied: cleaning, normalization, tokenization, and stemming. The results were improved by 4% using GA with CNN. Alzu'bi and Duwairi (2021) applied RNN to support multiple classes of classification models for dialects. They utilized 110000 sentences from the MADAR corpus, including Maghreb, Levantine, Gulf, and Iraqi dialects. Cotterell and Callison-Burch (2014) proposed Arabic dialects dataset collected from newspaper websites and Twitter, including five Arabic dialects: Levantine, Gulf, Egyptian, Iraqi, and Maghrebi. They utilized unigram, bigram, and trigram models and SVM and NB algorithms. NB with trigram achieved the best accuracy. In addition, Kwaik et al. (2018) proposed the Shami corpus for four Arabic dialects in Palestine, Jordan, Lebanon, and Syria. They explored the effects of pre-processing dialectal Arabic using n-gram and NB models. Various pre-processing steps were applied: cleaning, normalization, tokenization, and stemming. The results showed that NB recorded the highest accuracy. Alansari (2023) captured the semantic and phonological characteristics of dialects using CNN, and RNN. The proposed model comprises six stages: preprocessing, feature engineering, neural networks, optimization techniques, and evaluation methods. Shatnawi et al. (2023) applied different DL models: CNN-BiLSTM, Pooling-BiGRU, and AraBERT with different pre-trained word embedding FastText, AraVec, and AraBERT using a mix of a Katherine dataset that covers the dialects of eight nations and a NADI dataset acquired via Twitter that includes the dialects of twenty-one countries. In addition, they applied various data augmentation to handle unbalanced data. The results showed that models with AraBERT achieved the height performance.

Other researchers have suggested hybrid models, and attention mechanisms and transformer models to classify Arabic dialects. For example, Abdelazim et al. (2022) proposed a hybrid model (CNN-RNN) to classify three dialects: Gulf, Egypt, and Levantine. CNN-RNN, compared with NB, SVM, and CNN, recorded the best accuracy. Alsuwaylimi (2024) proposed two hybrid models that combined BiLSTM with CAMeLBERT and the second model that combined the BiLSTM model with AlBERT. In addition, the conducted dataset includes 121289 collected from comments from various social media platforms and classified into four Arabic dialects (Egyptian, Jordanian, Gulf, and Yemeni). Two models compared with different ML and DL models. Experiment results showed that two hybrid models recorded the best performance. Elaraby and Abdul-Mageed (2018) applied various ML models: SVM, RF, NB, LR, and different DL models: LSTM, GRU, Bi-LSTM, Bi-GRU, and Attention-BiLSTM using various word embedding. Results showed that attention-based BiLSTM work well compared to other models. Alghamdi et al. (2022) applied two transformer models, MARBERT and ARBERT, using two publicly available Arabic-dialect classification datasets such as AOC. They explored results for binary, three, and multi-class dialect classification. The results showed that MARBERT achieved higher performance than ARBERT.

Table 1 compares different models used in research. It outlines the methods, advantages, limitations, and datasets referenced in the studies.

**Table 1:** Comparison of existing work.

**References**	**Method**	**Advantages**	**Limitations**	**Dataset**
Lulu and Elnagar, 2018	LSTM	Proposing benchmark dataset	Applying the classical DL models Accuracy was lowest	AOC
Alsaleh and Larabi-Marie-Sainte, 2021	GA with CNN	Applying GA to optimize parameters of CNN	Applying the classical DL models Supporting text classification	Text classification
Alzu'bi and Duwairi, 2021	RNN	—	Applying single DL Using one dataset Obtaining the lowest accuracy	MADAR corpus
Cotterell and Callison-Burch, 2014	NB with Bi-gram	Proposing benchmark dataset	Applying ML models Using one dataset Obtaining the lowest accuracy	IADD
Kwaik et al., 2018	NB	Proposing benchmark dataset	Applying single model is NB Obtaining the lowest accuracy	Shami
Alansari, 2023	CNN and RNN	–	The results of the models have not been registered. Applying classical DL models	–
Shatnawi et al., 2023	AraBERT	Applying different wor-embedding Applying AraBERT Model	Obtaining the lowest accuracy	NADI
Abdelazim et al., 2022	RF	Proposing hybrid model	Applying classical DL models	Own
Elaraby and Abdul-Mageed, 2018	Attention BiLSTM	Proposing model based attention	Applying classical ML models. Using one dataset.	ADO
Alsuwaylimi, 2024	CAMeLBERT with BiLSTM	Proposing benchmark dataset Applying transformer models	No applying stacking models	ADO
Alghamdi et al., 2022	MARBERT	Applying transformer models	No applying stacking models	Own
Our work	Stacking-Transformer	Applying transformer to learn complex patterns in datasets.	–	IADD
	Stacking-Transformer	Applying generalization using stacking model based on two transformer models	–	Shami

## 3 Methodology

**Figure 1:**
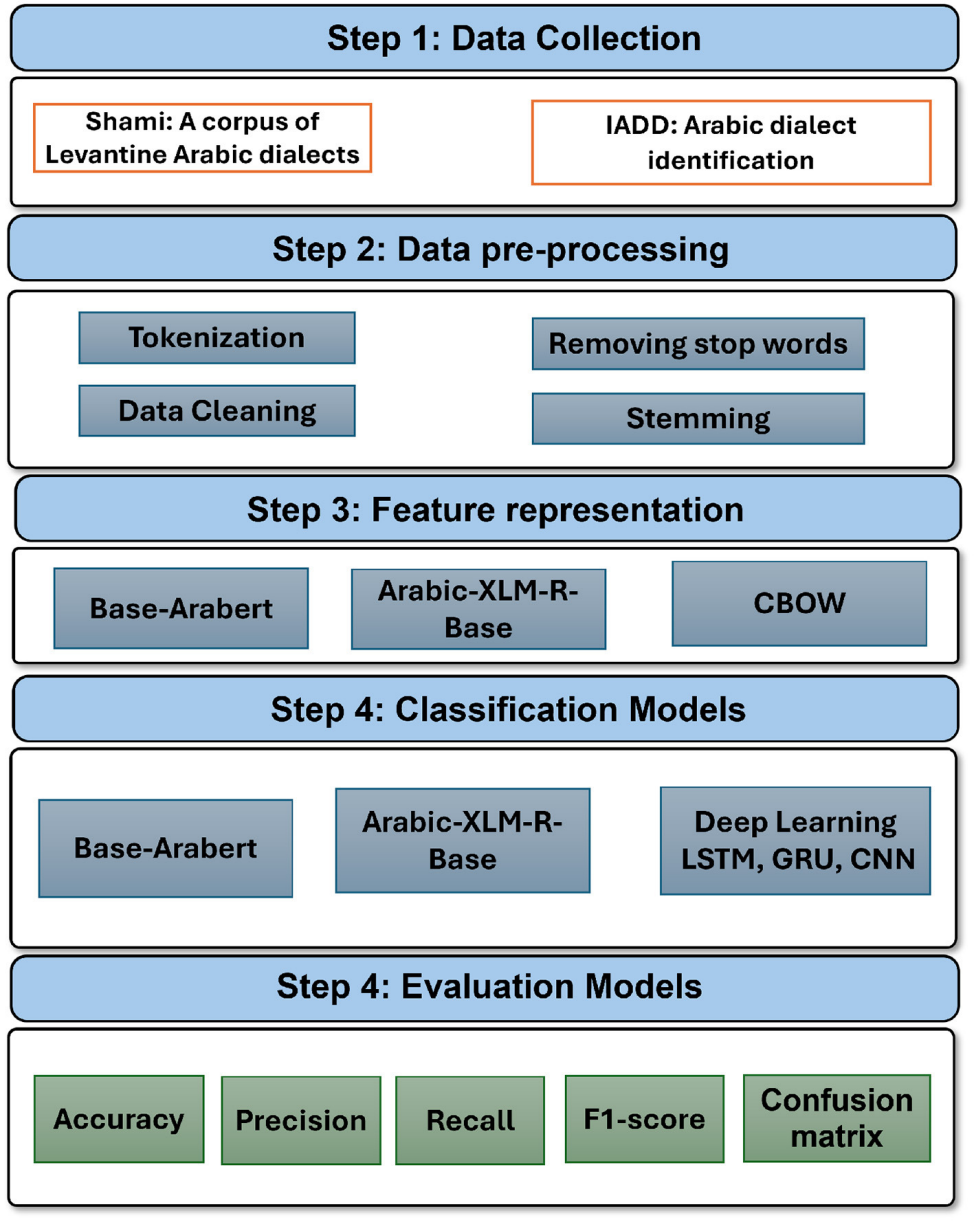
Arabic dialects classification framework.

Figure 1 shows the main steps of classifying Arabic dialects: Data collection, Data pre-processing, Classification models, feature representation methods, classification models, and evaluation models.

### 3.1 Datasets

Two benchmark Arabic dialect datasets are used for the experiment.

- Shami is a corpus of Levantine Arabic dialects (Kwaik et al., 2018) includes 66,245 rows with four dialect classes: Jordinian, Lebanees, Palestinian, and Syrian. The unbalanced dataset includes 37,758, 10,828, 10,642, and 7,017 rows for Syrian, Lebanese, Palestinian, and Jordanian, respectively.
- IADD is Arabic dialect identification (Zahir, 2022) is used and includes five dialects: Maghrebi (MGH), Levantine (LEV), Egypt (EGY), Iraq (IRQ), Gulf (GLF), and general. It was collected from tweets and Facebook.

### 3.2 Data pre-processing

Pre-processing the input data before starting to implement any model that processes text data is vital due to the various problems inherent, particularly in text data (Chai, 2023). Therefore, it is necessary to effectively rely on pre-processing the input text data to achieve a clear and accurate exploration of Arabic dialects based on stacked transformers. Data processing of the data aims to prepare and improve the quality of the input data to enhance the performance of the model. The four pillars of the pre-processing steps include Tokenization, data cleaning, stop word removal, and stemming (Kathuria et al., 2021). Carrying out these steps carefully will ultimately ensure that we obtain input data useful for accurately detecting the distinction between different Arabic dialects and obtaining a successful model in natural language processing tasks.

- Tokenization represents the first step in preparing textual data specifically, where the text is divided into smaller parts based on language-specific characteristics such as grammar and morphology (Khallaf, 2023). Tokenization comprises two types: word and sub-word Tokenization. In word tokenization, the result of this step is a set of separate words in addition to diacritics and linking marks. While Sub-word Tokenization is employed to handle out-of-vocabulary words and improve model robustness.
- Data Cleaning: The importance of this step lies in obtaining accurate data after removing irrelevant or confusing data that may hinder the performance of the model used. To accomplish this step, a normalization process must first be performed to convert different forms of the same word to its standard form, then deal with punctuation marks and special characters by removing or unifying them, especially those that do not affect the meaning (Berrimi, 2024). Also, deal with incorrect or incomplete data by neutralizing or removing them. After this step, we will ensure obtaining data of acceptable quality and consistency in its context, contributing to the model's success.
- Removing Stop Words enables the model to focus more on the main distinguishing features of dialects in the text. It thus improves the accuracy of the model in identifying and distinguishing them. Stop words represent a group of words that do not carry a critical or influential meaning in the context, and excluding them will positively reduce dimensions such as prepositions and articles (Khurana et al., 2023). These words are collected in a list to be excluded from the input data list.
- Stemming is a vital necessary process that reduces the expected complexity in the input data by converting words to their root form, which will allow better generalization when using the model to explore dialects (Farghaly and Shaalan, 2009). Many algorithms can be used during this step, some of which are designed specifically for the Arabic language due to its richness in morphology, which helps in grouping different morphological variants of a word. in this paper, stemming applies using Arabic-specific stemming algorithms to handle the morphological richness of Arabic. The algorithms are chosen carefully to prevent mistakes like confusing words with the same root but distinct meanings. In the context of Arabic dialects, this guarantees the results' validity and correctness.

### 3.3 Dataset splitting

Each dataset is split into a 75% training set and a 25% testing set. The split preserves enough data for objective assessment while guaranteeing reliable model training. Methods for feature representation are customized for the datasets.

### 3.4 Feature representation methods

While conventional DL models employed CBOW for word embeddings, transformer-based models like Bert-Base-Arabertv02 and Dialectal-Arabic-XLM-R-Base are utilized to generate high-quality contextual embeddings.

- Word2Vec is a widely used technique for learning word embeddings from large volumes of textual data (Karani, 2018). This approach generates embeddings by considering the context in which words appear, enabling the representation of words in a continuous vector space that captures semantic relationships (Karani, 2018). Word2Vec effectively reduces the dimensionality of the word space while preserving meaningful relationships between words, offering a computationally efficient solution for processing language data (Dwivedi and Shrivastava, 2017). One variant of Word2Vec is the Continuous Bag-of-Words (CBOW) model (Sivakumar et al., 2020). CBOW predicts a target word based on its surrounding context words within a fixed-size window. The model is designed to maximize the probability of correctly predicting the target word, leveraging contextual information to enhance its learning capability (Melamud et al., 2016).
- Bidirectional Encoder Representations from Transformers (BERT) is the open-source transformer-based model that is renowned for its ability to model contextual relationships among words within a sentence through self-attention mechanisms (Vig, 2019). Thanks to this architecture, BERT excels at capturing contextual information and long-range dependencies (Wu et al., 2021). BERT profoundly comprehends linguistic subtleties by being pre-trained on vast volumes of unlabeled text data utilizing two unsupervised tasks. Namely, masked language modeling (MLM) and next sentence prediction (NSP) (Kryeziu and Shehu, 2022). In MLM, words from the input text are randomly masked. BERT is subsequently taught to predict these masked words through analysis of the surrounding context (Devlin et al., 2018). BERT can improve its skills on particular tasks by employing relatively more minor labeled datasets, even when pre-trained on massive quantities of data (Devlin et al., 2018). Bert-base-Arabic refers to the BERT model specially trained on the Arabic language, offering pre-trained representations that encapsulate both syntactic and semantic nuances of Arabic words (Chouikhi et al., 2021). This model accepts Arabic text as input and outputs contextualized word representations, which can be further refined using task-specific training data or directly utilized in downstream NLP tasks (Peters et al., 2019).
- Dialectal Arabic XLM-R Base represents a multilingual transformer model customized to comprehend and interpret several Arabic dialects (Khalifa et al., 2021). An expansion of the BERT architecture called the Cross-lingual Language Model (XLM-R) is intended to function with various languages, including dialects and languages with limited resources (Boudad et al., 2023). This transformer can cope with multiple Arabic dialects alongside other languages since it has been taught on many datasets. Conversational agents can be upgraded to more effectively comprehend and respond to dialectal Arabic more Base using the dialectal Arabic XLM-R Base (Joshi et al., 2024).
  By refining the translations between dialects and standard Arabic, it will be feasible to assess the thoughts and feelings expressed across dialects on social media or in reviews. Built on top of the XLM-R architecture, the Dialectal Arabic XLM-R Base architecture preserves the transformer architecture's scalability and efficacy while being tailored for the complex structure of dialectal Arabic. The model can figure out the word order in a sentence by mapping input tokens to dense vectors and then adding positional information to token embeddings (Qwaider and Abu Kwaik, 2022). Multi-head Self-Attention has been included to allow the model to concentrate on various segments of the input stream concurrently, thereby capturing contextual linkages. A feedforward network processes each attention output before applying it separately to each point. Improves training stability and convergence via normalizing the inputs to each layer (Berrimi, 2024).

### 3.5 Deep learning models

GRU, LSTM, and CNN are used for DL models.

- GRU is a recurrent architecture with update and reset gates intended to handle sequential data. The update gate controls how much past knowledge remains intact, whereas the Reset gate governs whether earlier data is forgotten (Dey and Salem, 2017). GRU has a hidden state that blends the current input and the prior hidden state, permitting information to flow through time. GRU is appropriate for tasks that need time series data and sequential information, such as language modeling and machine translation (Zargar, 2021). It is beneficial for determining context in textual data.
- LSTM is a more complicated recurrent architecture having forgotten, input, and output gates suitable for learning long-term dependencies (Okut, 2021). The forget gate regulates what information to exclude from the cell state, whereas the input gate determines what latest data to store in the cell state. The output gate determines which information to output based on the cell state (Okut, 2021). The cell state sustains long-term dependencies, allowing gradients to propagate throughout multiple time steps. LSTM can be utilized for text synthesis, machine translation, and speech recognition (Van Houdt et al., 2020). Also, it is competent at predicting potential outcomes using historical and time series data.
- CNN is a type of neural network that comprises convolutional and pooling layers, which help generate features from spatial data. CNN leverages convolution processes to extract features from input data, often images or sequences (Pinaya et al., 2020). It mitigates the spatial dimensions via down-sampling while maintaining the most significant features and then connects the pooled information to the output layer for classification or regression. CNN is frequently implemented for object detection and image segmentation. It also works for sentiment analysis and spam identification since it treats text data as a series (Bhuvaneshwari et al., 2021).

### 3.6 Proposed model

**Figure 2:**
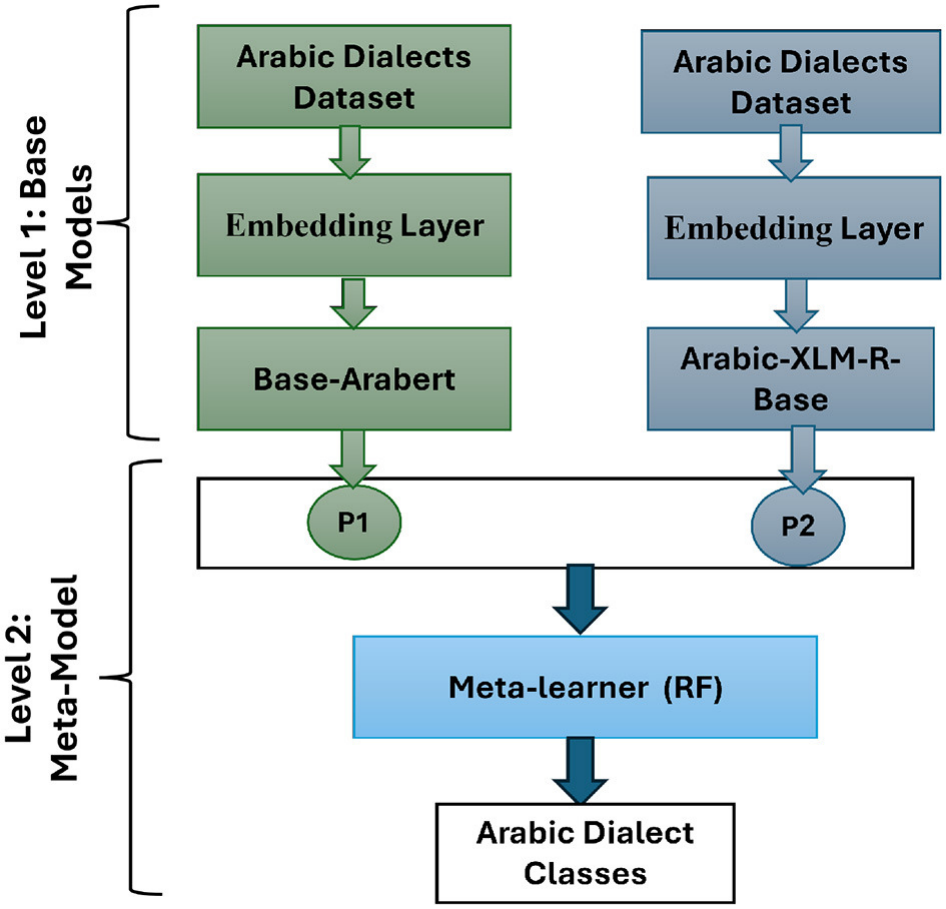
Proposed model.

By integrating the strengths of various models, the stacking approach reflects a wide range of linguistic features, resulting in improved dialect detection. Figure 2 shows the central architecture's two levels. Level 1 provides the base models with the two transformers that produce class probabilities for training and testing datasets. The second level serves as a meta-learner, which is trained using Level 1's outputs, resulting in enhanced classification performance.

In Level 1, class probabilities are generated by the two transformer models for the training and testing sets and are stored in the stacking training and stacking testing datasets, respectively. In level 2, RF as a meta-learner is trained by stacking training and evaluated by stacking testing to get the final classification decision. RF is an ensemble technique that uses several decision trees during training and combines their outputs for more accurate and stable predictions (Feng et al., 2015).

### 3.7 Models evaluation

The F1-score, Accuracy, Precision, and Recall metrics are used to assess the models. Where TN indicates the aggregate amount of accurate negative predictions, FP is the total number of false positive estimations, while FN stands for the overall number of false negative predictions.


(1) $$\begin{array}{l} Accuracy=\frac{TP+TN}{TP+FP+TN+FN} \end{array}$$

(2) $$\begin{array}{l} Recall=\frac{TP}{TP+FN} \end{array}$$

(3) $$\begin{array}{l} Precision=\frac{TP}{TP+FP} \end{array}$$

(4) $$\begin{array}{l} F1-score=2\cdot \frac{precision\cdot recall}{precision+recall} \end{array}$$

## 4 Results and discussion

We applied different experiments using various models and two datasets to prove that the Stacking-Transformer model achieved the best performance compared to other models.

### 4.1 Experimental setup

The experiment was conducted on a laptop with an Intel Core i7 10750H and 16GB memory. The execution environment for the training and validation of the networks was set to a single GPU: Nvidia GeForce GTX 1650 with 4GB VRAM. The models were evaluated by two datasets: Shami with four classes (Jordinian, Lebanees, Palestinian, and Syrian) and IADD with five classes (EGY, GLF, LEV, MGH, and general). Base-Arabert and Dialectal-Arabic-XLM-R-Base are used as feature representations for transformer models, and CBOW is used for DL models. The datasets are split into 75% training set and 25% testing set and the number of rows in each dataset is shown in Table 2. The setting of parameters of models are presented in Table 3.

**Table 2:** The number of rows in each dataset.

**Datasets**	**Labels**	**Training set**	**Testing set**	**Total**
Shami	Syrian	28,318	9,440	37,758
	Lebanees	8,121	2,707	10,828
	Palestinian	7,981	2,661	10,642
	Jordinian	5,263	1,754	7,017
	Total	49,683	16,562	66,245
IADD	LEV	65,605	21,864	87,469
	MGH	21,037	7,076	28,113
	GLF	5,011	1,671	6,682
	EGY	3,626	1,209	4,835
	general	1,873	625	2,498
	Total	97,152	32,445	129,597

**Table 3:** Setting of parameters.

**Models**	**Parameters**	**Specifications**
LSTM	Number of nodes	200
	Dropout	0.2
	Activation function	Relu
	Optimizer	Adam
	Loss function	CrossEntropyLoss
GRU	Number of nodes	200
	Dropout	0.2
	Activation function	Relu
	Optimizer	Adam
	Loss function	CrossEntropyLoss
CNN	Filter size	3x3
	Kernel size	4
	Dropout	0.2
	Optimizer	Adam
	Loss function	CrossEntropyLoss
Bert-Base-Arabertv02	Number of transformer layers	12
	Hidden Size	768 dimensions
	Attention Heads	12 per layer
	Optimizer	Adam
	Loss function	CrossEntropyLoss
	Dropout rate	0.1
Dialectal-Arabic-XLM-R-Base	Number of transformer layers	12
	Hidden Size	768 dimensions
	Attention Heads	12
	Optimizer	Adam
	Loss function	CrossEntropyLoss

### 4.2 Results

Two subsections present the results of Shami and IADD based on precision, recall, F1-score in each class, and confusion matrices. Furthermore, the average accuracy, precision, recall, and F1-score of each dataset is presented.

#### 4.2.1 Proposed model performance in Shami dataset

The results of models based on precision, recall, and F1-score for different classes: Jordinian, Lebanees, Palestinian, and Syrian as shown in Table 4. We can see that GRU, LSTM, and CNN score the lowest in performance compared to transformer models because CNN models focus on local feature extraction but fail to capture complex, long-term relationships. GRU and LSTM handle sequential data, and they have limits to capturing long-term dependencies, especially with large datasets. Transformer-based models leverage self-attention mechanisms to learn both local and global patterns in parallel dynamically, and capture long-term dependencies.

**Table 4:** Proposed model performance in Shami dataset.

**Approaches**	**Models**	**Classes**	**Precision**	**Recall**	**F1-score**
DL models	GRU	Jordinian	60.84	55.53	58.06
		Lebanees	77.05	77.39	77.22
		Palestinian	69.22	73.28	71.19
		Syrian	91.28	91.13	91.21
	LSTM	Jordinian	62.25	50.40	55.70
		Lebanees	73.45	75.03	74.23
		Palestinian	72.37	65.54	68.78
		Syrian	87.59	92.48	89.97
	CNN	Jordinian	62.25	50.40	55.70
		Lebanees	73.45	75.03	74.23
		Palestinian	72.37	65.54	68.78
		Syrian	87.59	92.48	89.97
The transformer model	Base-Arabert	Jordinian	80.16	61.52	69.61
		Lebanees	84.64	79.61	82.05
		Palestinian	77.64	82.60	80.04
		Syrian	92.07	95.96	93.98
	Arabic-XLM-R-Base	Jordinian	79.77	60.03	68.51
		Lebanees	84.49	79.09	81.70
		Palestinian	77.34	82.60	79.88
		Syrian	91.82	95.96	93.85
The proposed model	Stacking-Transformer	Jordinian	80.16	61.52	69.61
		Lebanees	84.64	79.61	82.05
		Palestinian	77.64	82.60	80.04
		Syrian	92.07	95.96	93.98

The following summarizes the results models with Jordinian record the lowest rates compared to other classes. Models with Syrian class record the highest rate. GRU with Syrian has the highest precision, recall, and F1-score at 91.28, 91.13, and 91.21, respectively. LSTM with Syrian records 91.13 recall higher than GRU. GRU with Lebanees class has the second-highest performance compared to CNN and LSTM with 77.05 precision and 77.22 with F1-score. CNN and LSTM with Lebanees and Palestinian have the same approximate results. Base-Arabert and Arabic-XLM-R-Base with Syrian class record the same recall at 95.96. Both record the same precision, recall, and F1-score at 84.49, 79.09, and 81.70, respectively with Lebanees class. Stacking-Transformer records the highest performance in all classes compared to other models. The best precision, recall, and F1-score are achieved by Stacking-Transformer with Syrian, at 92.07, 95.96, and 93.98, respectively.

**Figure 3:**
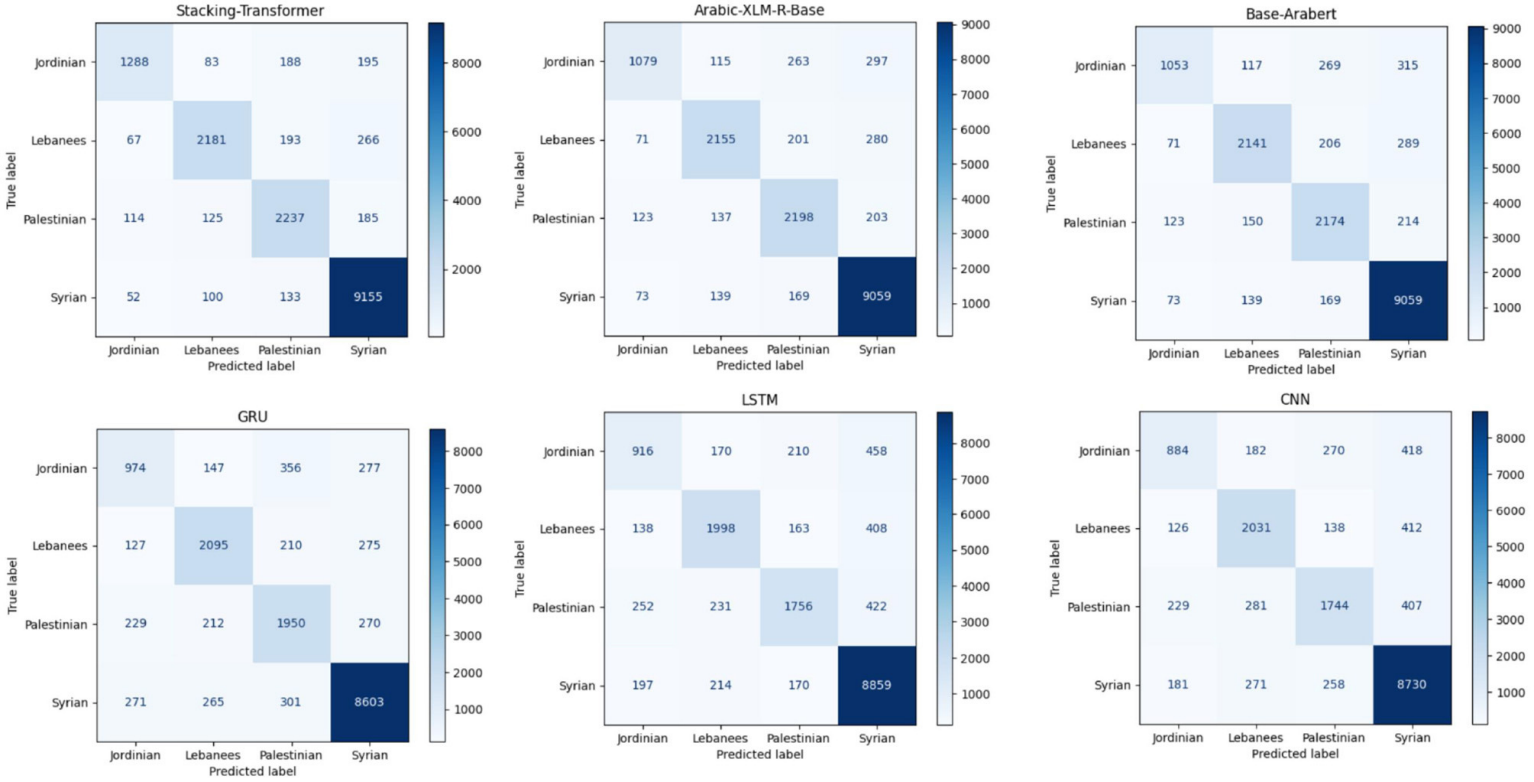
Confusion matrices of models for Shami.

Figure 3 comprises six confusion matrices, each of which shows how various models performed in a classification exercise aimed at classifying data into one of four groups: Syrian, Palestinian, Lebanese, or Jordanian. Four groups are created from the models: Syrian, Palestinian, Lebanese, and Jordanian. Darker colors indicate higher counts. The color intensity in each confusion matrix reflects the number of samples sorted into each class. Classifying the Syrian category appears to be generally easier across all models, but the Palestinian and Jordanian categories are more difficult.

#### 4.2.2 Proposed model performance in IADD dataset

**Figure 4:**
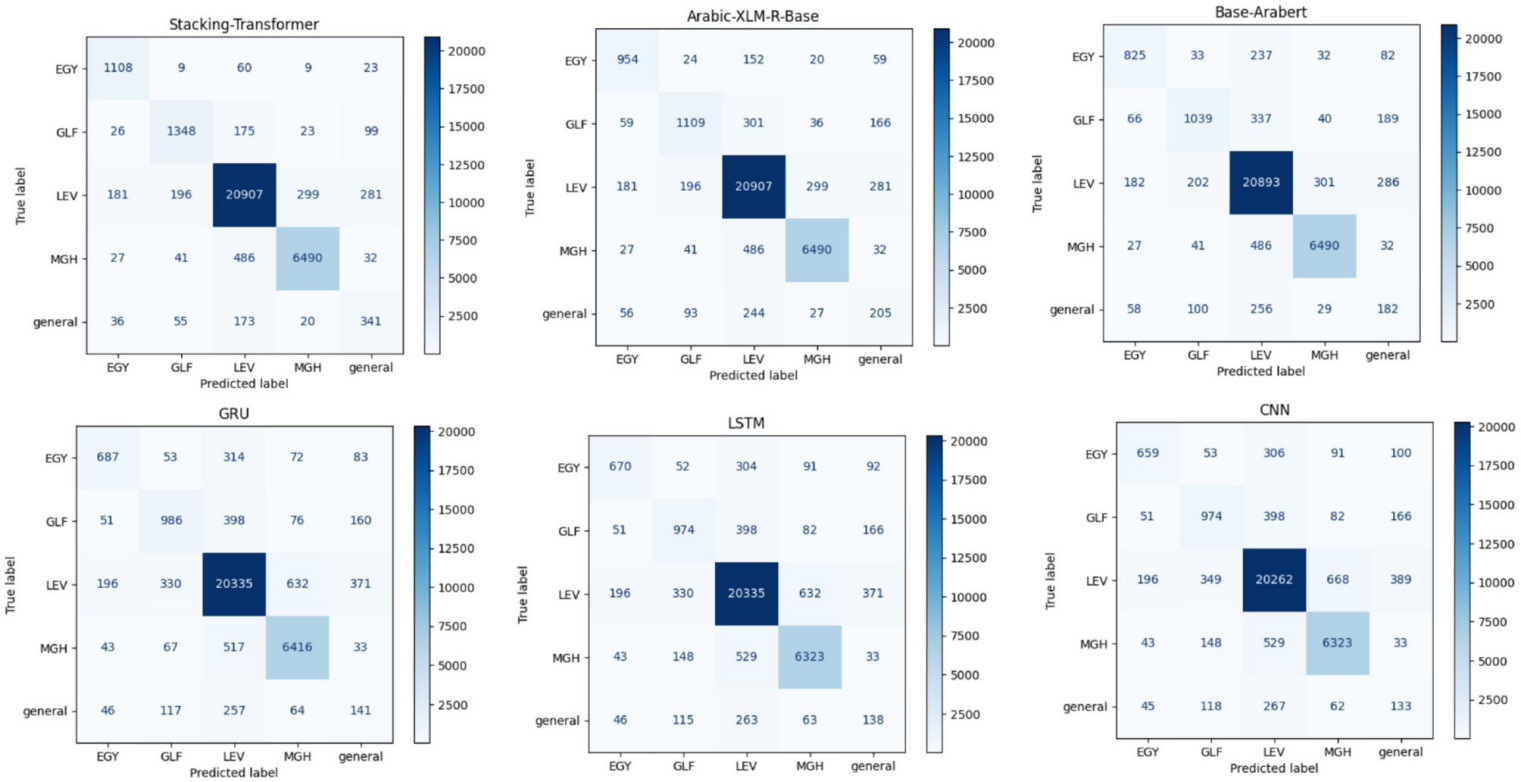
Confusion matrices of models for IADD.

Table 5 presents the precision, recall, and F1-score for different classes: EGY, GLF, LEV, MGH, and general for each model. The best precision, recall, and F1-score are achieved by GRU and LSTM with LEV, at 93.19, 93.01, and 93.10, respectively. GRU and LSTM general EGY record the same approximate results. In comparison to CNN and LSTM, GRU with MGH class has the second-highest precision (90.67) and F1-score (89.51). Of all the models based on each class, CNN yields the lowest results. Base-Arabert with GLF records precision, recall, and F1-score at 73.43, 62.18, and 67.34, respectively, compared to DL models. Arabic-XLM-R-Base with LEV and MGH classes records the same precision at 94. The stacking Transformer records the highest performance in all classes compared to other models. The best precision, recall, and F1-score are achieved by Stacking-Transformer with LEV, at 95.90, 95.6, and 95.76, respectively. Also, it has significant performance in the general class compared to other models. Figure 4 comprises six confusion matrices, each of which shows how various models performed in a classification exercise aimed at classifying data into one of five groups: EGY, GLF, LEV, MGH, and general. Darker colors indicate higher counts. The color intensity in each confusion matrix reflects the number of samples sorted into each class.

**Table 5:** Performance of proposed model in Shami dataset.

**Approches**	**Models**		**Precision**	**Recall**	**F1-score**
DL models	GRU	EGY	67.16	56.82	61.56
		GLF	63.49	59.01	61.17
		LEV	93.19	93.01	93.10
		MGH	88.37	90.67	89.51
		general	17.89	22.56	19.96
	LSTM	EGY	66.60	55.42	60.50
		GLF	60.16	58.29	59.21
		LEV	93.16	93.01	93.08
		MGH	87.93	89.36	88.64
		general	17.25	22.08	19.37
	CNN	EGY	66.30	54.51	59.83
		GLF	59.32	58.29	58.80
		LEV	93.11	92.67	92.89
		MGH	87.50	89.36	88.42
		general	16.20	21.28	18.40
The transformer model	Base-Arabert	EGY	71.24	68.24	69.71
		GLF	73.43	62.18	67.34
		LEV	94.07	95.56	94.81
		MGH	94.17	91.72	92.93
		general	23.61	29.12	26.07
	Arabic-XLM-R-Base	EGY	74.71	78.91	76.75
		GLF	75.80	66.37	70.77
		LEV	94.64	95.62	95.13
		MGH	94.44	91.72	93.06
		general	27.59	32.80	29.97
The proposed model	Stacking-Transformer	EGY	80.41	91.65	85.66
		GLF	81.75	80.67	81.20
		LEV	95.90	95.62	95.76
		MGH	94.87	91.72	93.27
		general	43.94	54.56	48.68

#### 4.2.3 Discussion

**Figure 5:**
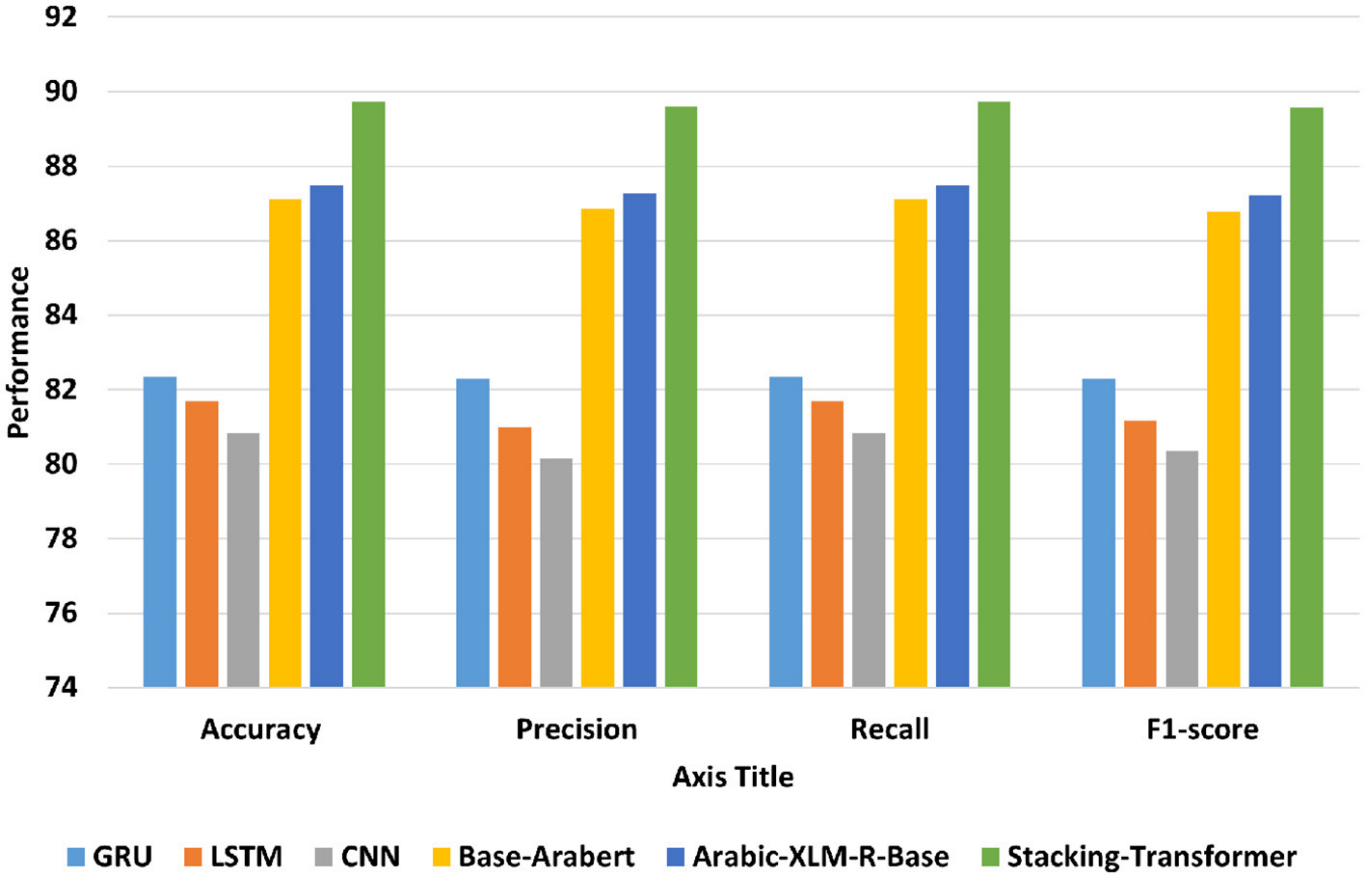
Average accuracy, precision, recall, and F1-score of models for Shami.

Transformer models have achieved state-of-the-art performance across various tasks compared to traditional DL models for several key reasons the self-attention mechanism in transformers allows them to consider all parts of the input sequence simultaneously. This enables the model to capture long-range dependencies more effectively than traditional recurrent, which are typically limited by sequential processing or fixed-size filters. Figure 5 shows the average accuracy, precision, recall, and F1-score of DL models, transformer models, and the proposed model (Stacking-Transformer) for classifying Syrian, Lebanees, Palestinian, Jordinian. From the table, transformer models record the best performance compared to deep learning models and improve results by improving results above 5%. The transformer models have the attention that can capture long-range dependencies more effectively than DL models. Arabic-XLM-R-Base has the highest performance compared to Base-Arabert, LSTM, GRU, and CNN with accuracy = 87.495, precision = 87.278, recall = 87.495, and F1-score = 87.209. CNN has the worst all measures with 80.842 of accuracy and 80.363 of F1-score. Stacking-Transformer has the highest performance in all rates with 89.73 of accuracy and 89.574 of f1-score.

**Figure 6:**
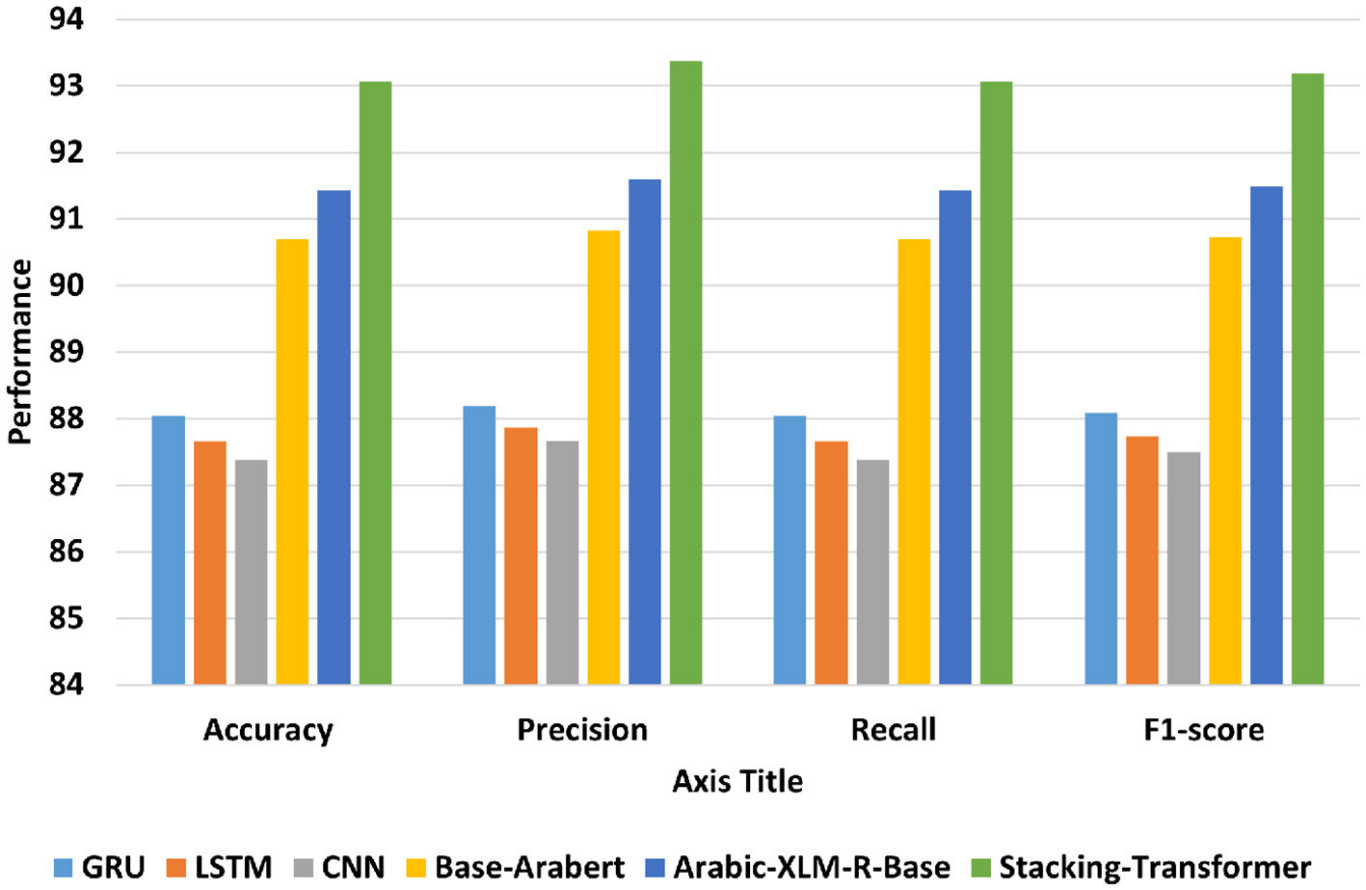
Average accuracy, precision, recall, and F1-score of models for IADD.

Figure 6 shows the average accuracy, precision, recall, and F1-score of DL models, transformer models, and the proposed model (Stacking-Transformer) for classifying EGY, GLF, LEV, MGH, and general. From the table, transformer models record the best performance compared to DL models and improve results by improving results above 2%. Arabic-XLM-R-Base has the highest performance compared to Base-Arabert, LSTM, GRU, and CNN with accuracy = 91.432, precision = 91.595, recall = 91.432, and f1-score = 91.485. CNN has the worst of all measures with 87.382 of accuracy and 87.492 of F1-score. Stacking-Transformer has the highest performance in all rates with 93.062 of accuracy and 93.184 of f1-score, and improve performance by 2 compared to Arabic-XLM-R-Base.

### 4.3 Comparison of the proposed model with existing work

Table 6 compares our work with the state-of-the-art based on dataset and results. The proposed model, Stacking-Transformer, is based on two transformer models as the baseline and an RF as the meta-learner. It achieves the highest accuracy due to the advantages of the attention mechanism in the transformer, which extracts long dependencies between text, and the generalization capability of stacking models. For IADD, Stacking-Transformer recorded the highest accuracy at 93.062 compared to NB with Bi-gram, which was recorded at 70 in Cotterell and Callison-Burch (2014). For Shami, the Stacking-Transformer recorded the highest accuracy at 89.73 compared to NB in Kwaik et al. (2018). For ADO as a subset of Shami, LSTM was used in Lulu and Elnagar (2018) and recorded 71.4 accuracy. In Elaraby and Abdul-Mageed (2018), Attention BiLSTM recorded 87.81 of accuracy. CAMeLBERT with BiLSTM was recorded at 87.

**Table 6:** Comparison with existing work and the proposed models based on models and performance.

**References**	**Methods**	**Results**	**Datasets**
Lulu and Elnagar, 2018	LSTM	71.4	AOC
Cotterell and Callison-Burch, 2014	NB with Bi-gram	87.00	IADD
Kwaik et al., 2018	NB	70	Shami
Elaraby and Abdul-Mageed, 2018	Attention BiLSTM	87.81	ADO
Alsuwaylimi, 2024	CAMeLBERT with BiLSTM	87	ADO
Our work	Stacking-Transformer	93.062	IADD
	Stacking-Transformer	89.73	Shami

### 4.4 Implication and challenges

The proposed investigation has important ramifications for expanding NLP applications and improving Arabic dialect identification. The paper shows improved accuracy, precision, and recall in dialect classification via a hybrid stacking model that incorporates the advantages of transformer designs such as Dialectal-Arabic-XLM-R-Base and Bert-Base-Arabertv02. Given the increasing amount of dialectal material on social media and other platforms, the development fills a significant gap in NLP for managing the linguistic variety of Arabic. The model's cross-dialect generalization establishes a new standard for datasets like Shami and IADD, providing a solid basis for further study and advancement. Additionally, the study has practical applications, such as enhancing conversational AI, sentiment analysis, and machine translation systems to better interpret a variety of complex language inputs.

The paper points out several challenges, including substantial differences in syntax, vocabulary, and semantics between regional dialects and Modern Standard Arabic (MSA) pose a difficult obstacle for models to overcome, especially when generalizing across underrepresented dialects; data imbalance, as seen in the Shami dataset, makes this problem worse and restricts the performance of models on less represented classes, like Jordanian dialects; and the computational demands of training and fine-tuning stacked transformer models demand a significant amount of resources, which may limit accessibility for researchers with limited financial resources. Challenges with scalability and practical implementation also exist, especially for real-time applications that may encounter resource constraints and latency, such as chatbots and virtual assistants. Tokenization, stemming, and stop-word deletion are examples of preprocessing processes that increase complexity since they might not adequately capture the subtle differences present in dialectal Arabic. Even if the model produces state-of-the-art results on certain datasets, there is still a need for more research in generalizing Arabic dialects or languages with equally complex linguistic patterns.

## 5 Conclusion

In this paper, we introduced a unique stacking model that combines two potent transformer models, Bert-Base-Arabertv02 and Dialectal-Arabic-XLM-R-Base, with a meta-learner to improve the categorization of Arabic dialects. The model formed involved two levels: base models and meta-learners. Within level one, the two transformer models yield class probabilities for the training and testing sets, which are retained in stacking training and stacking testing, respectively. Level 2 meta-learners with machine learning models are trained and tested using stacking. The stacking model has been contrasted against multiple models, including LSTM, GRU, CNN, and two transfer models with distinct word embedding. Models were assessed on two benchmark datasets to classify four and five dialects of Arabic, featuring various evaluation matrices, including accuracy, precision, recall, F1-score, and confusion matrix. The results proved that the stacking model outperformed single-model techniques. The proposed model addressed a wider spectrum of linguistic traits, allowing for more accurate generalization across different varieties of Arabic. Shami dataset testing reveals that the Stacking-Transformer outperforms all other models in accuracy, precision, recall, and f1-score, with 89.73, 89.596, and 89.574, respectively. For IADD, Stacking-Transformer outperforms other models in all rates, with 93.062 accuracy, 93.368 precision, 93.062 recall, and 93.184 F1-score. In the future, we will concentrate on developing this method to handle other dialects and investigating whether it can be used in other low-resource languages with comparable linguistic complexity.

## Data Availability

The original contributions presented in the study are included in the article/supplementary material, further inquiries can be directed to the corresponding author.
